# Building flexibility and managing complexity in community mental health: lessons learned in a large urban centre

**DOI:** 10.1186/s12888-018-1597-y

**Published:** 2018-01-24

**Authors:** Vicky Stergiopoulos, Dima Saab, Kate Francombe Pridham, Anjana Aery, Arash Nakhost

**Affiliations:** 1 Centre for Urban Health Solutions, Li Ka Shing Knowledge Institute, St. Michael’s Hospital, 209 Victoria Street, Toronto, ON M5B 1W8 Canada; 2 Centre for Addiction and Mental Health, 100 Stokes Street, Toronto, ON M6J 1H4 Canada; 3 Department of Psychiatry, University of Toronto, 250 College Street, 8th Floor, Toronto, ON M5T 1R8 Canada; 4 Mental Health Services, St. Michael’s Hospital, 30 Bond Street, Toronto, ON M5B 1W8 Canada

**Keywords:** Mental health, Complex needs, Qualitative evaluation, Community mental health, Implementation, Flexible assertive community treatment

## Abstract

**Background:**

Across many jurisdictions, adults with complex mental health and social needs face challenges accessing appropriate supports due to system fragmentation and strict eligibility criteria of existing services. To support this underserviced population, Toronto’s local health authority launched two novel community mental health models in 2014, inspired by Flexible Assertive Community Team principles. This study explores service user and provider perspectives on the acceptability of these services, and lessons learned during early implementation.

**Methods:**

We purposively sampled 49 stakeholders (staff, physicians, service users, health systems stakeholders) and conducted 17 semi-structured qualitative interviews and 5 focus groups between October 23, 2014 and March 2, 2015, exploring stakeholder perspectives on the newly launched team based models, as well as activities and strategies employed to support early implementation. Interviews and focus groups were audio recorded, transcribed verbatim and analyzed using thematic analysis.

**Results:**

Findings revealed wide-ranging endorsement for the two team-based models’ success in engaging the target population of adults with complex service needs. Implementation strengths included the broad recognition of existing service gaps, the use of interdisciplinary teams and experienced service providers, broad partnerships and collaboration among various service sectors, training and team building activities. Emerging challenges included lack of complementary support services such as suitable housing, organizational contexts reluctant to embrace change and risk associated with complexity, as well as limited service provider and organizational capacity to deliver evidence-based interventions.

**Conclusions:**

Findings identified implementation drivers at the practitioner, program, and system levels, specific to the implementation of community mental health interventions for adults with complex health and social needs. These can inform future efforts to address the health and support needs of this vulnerable population.

**Electronic supplementary material:**

The online version of this article (10.1186/s12888-018-1597-y) contains supplementary material, which is available to authorized users.

## Background

Across jurisdictions, individuals with complex health, mental health and social needs face multiple barriers to accessing appropriate, integrated services and supports due to system fragmentation, strict eligibility criteria of existing services, stigma and discrimination.

In western countries, individuals with serious mental illness (SMI) can access Assertive Community Treatment (ACT) or Intensive Case Management (ICM) services. Both ACT and ICM models have been studied in a wide range of contexts and for various subpopulations of adults with SMI [1–6]. ACT is a well-defined, team-based approach to care, with strong evidence in its favour [3, 5]. Its strict eligibility criteria, however, may exclude a subset of individuals with mental disorders and high support needs (e.g. individuals with primary substance use, or personality disorders) [7]. Furthermore, with few options for seamless participant transitions to lower levels of support during periods of stability, there are long waitlists for ACT services in several jurisdictions [8]. In contrast to ACT, with its well-developed fidelity criteria, ICM tends to be more variable in nature and implementation, yielding mixed findings in the evaluative literature [9, 10]. While ACT teams directly provide needed services to adults with SMI and high service utilization, ICM interventions deliver these same services in collaboration with other local service-providers [9], and generally provide a lower level of support to a broader group of adults with SMI.

To address some of these challenges, Flexible Assertive Community Treatment (FACT), a model that blends aspects of ACT and ICM services within a single team, emerged in the Netherlands in the past 10- years, and has been well described and adopted elsewhere [11–14]. Within a FACT team, service users retain the relationships with their care manager (nurse/social worker), their psychiatrist and other team members such as the peer expert, while stepping-up or down to higher (ACT-like) or lower (enriched ICM) levels of support as needed over time. As FACT eligibility criteria are more flexible than those of traditional ACT team criteria, ACT-ineligible individuals may access high levels of support services if needed. Early evaluations of the FACT model, relying primarily on observational studies and administrative data, have been promising, suggesting improvements in adherence rates, reduction in unmet needs and improved quality of life [12, 15–19].

This study describes the early implementation of two local community mental health teams, inspired by FACT principles, to address the needs of adults with complex health, mental health and social needs in Toronto, Canada’s largest urban centre. The local service delivery context, and guidelines from the local health authority necessitated departures from full replication of FACT.

### Service delivery context

Assertive Community Treatment (ACT) teams in the province of Ontario, Canada have been systematically implemented since 1998, with 79 ACT teams currently in operation [20]. Though Toronto has a high concentration of services, including ACT, individuals with complex health and social needs such as intellectual and developmental disabilities, traumatic brain injury, co-morbid substance use conditions, and co-morbid personality disorders, continue to face barriers in accessing community supports of high intensity. Similar to other jurisdictions, Ontario ACT standards prioritize adults with schizophrenia and bipolar affective disorder, creating access challenges for those not meeting diagnostic eligibility criteria or not having repeated and lengthy hospitalizations [21]. With ACT team wait lists averaging more than a year [22], local ICM services are often asked to step in while they are not resourced to serve individuals with complex health and social needs requiring more intensive interventions.

To address these challenges, Toronto’s local health authority launched two “Integrated Service (IS) Teams”, drawing from elements of FACT, in 2014. Although previous reviews have synthesized the factors that affect implementation of programs in general into a conceptual model [23, 24], and Fixsen et al. (2005) have developed the National Implementation Research Network (NIRN) framework for active implementation [25], these models are still evolving. Furthermore, research on how implementation unfolds in community based mental health services and systems at various stages of implementation is scant [26–28], including research on implementing ACT, or FACT [17, 18, 29, 30].

### Study aims

This study aims to explore service user and provider perspectives on the acceptability of the IS team models, and to identify early implementation drivers. This information may be useful for future efforts to address the needs of adults with complex health, mental health and social needs in other jurisdictions facing similar challenges.

## Methods

### The integrated service teams: introducing team and organizational flexibility

Both Integrated Service (IS) teams, referred to throughout this paper as the “East” and “South” team, for their respective geographical areas in the city, were multidisciplinary, recovery oriented, and targeted individuals with complex needs (Table 1). Eligibility criteria focused on the social circumstances of service users, such as homelessness, criminal justice involvement, and service engagement, rather than strict diagnostic or past health service use criteria.

**Table 1:** East and South Team comparisons with ACT and FACT models

	ACT Standards in Ontario, Canada^a^	East Team	South Team	FACT in the Netherlands^b^
Target Population	Adults with serious mental illness (SMI) that seriously impairs their social functioning. Priority given to adults with schizophrenia, other psychotic disorders and bipolar disorder.	Adults meeting ACT admission criteria as well as adults experiencing complex mental, physical and social needs (including homelessness, criminal justice involvement, developmental and substance use disorders), with high utilization of acute care services and poor track record of system engagement.	Youth and adults (16-65 years) with complex mental, physical and emotional needs (including trauma, developmental and personality disorders), with high utilization of acute care services and poor track record of system engagement.	All patients with serious mental illness (SMI) in a particular district or region (including major axis I and severe limitations in social functioning).
Team size	60-100	190-208	90-100	220-250
Caseload	Urban - 1:10	1:13	1:13	1:20
Catchment area	1:200, 000	1:130,000-150,000	1:130,000-150,000	1: 40,000 -50,000
Clinical Human Resources	11 FTE clinical staff excluding team psychiatrist:	20.5 FTE clinical staff excluding team psychiatrists:	11 FTE clinical staff excluding consulting psychiatrist:	11 to 14 FTE clinical staff excluding team psychiatrist:
	1 FTE team coordinator; 3 FTE registered nurses; 1 FTE social worker; 1 FTE occupational therapist;1 FTE addiction specialist; 1 FTE vocational specialist;1 FTE peer support specialist; 2 FTE case managers; 0.8 FTE psychiatrist	1 FTE team coordinator;1 FTE psychologist; 5 FTE psychiatric nurses;2 FTE social workers; 2 FTE occupational therapists; 1.5 FTE peer support specialist; 1 FTE personal support worker; 1 FTE addiction specialist;1 FTE rehabilitation therapist;1 FTE vocational specialist;4.0 FTE case managers;1.3 FTE psychiatrists	2.0 FTE care coordinators; 1.0 FTE nurse practitioner, 2.0 FTE behavioral therapists; 0.5 FTE personal support worker; 2.0 FTE social workers; 2.0 FTE nurses; 1.0 FTE registered practical nurse; access to consulting psychiatrist	5 to 8 FTE psychiatric nurses;1 FTE psychologist;1 FTE employment specialist;1 FTE peer support worker;1 FTE social worker; 2 FTE addiction specialists; 1.0 FTE psychiatrist
Team rounds	Daily meeting to discuss clients in crisis or update the team on ongoing issues.	Using the FACT board for daily morning meeting for 30–50 clients requiring daily attention.	Weekly meetings.	Using the FACT board for daily meetings for 20-30 clients requiring daily attention.
Team Vision	ACT services to clients who have not benefited from traditional out-patient programs.	Recovery focused ACT and ICM services within the same team. EBM interventions provided as core component of team function.	Recovery focused ICM level multidisciplinary support to clients, facilitating smooth care transitions within the organization as client support needs change. Select EBM interventions are provided.	Recovery focused ACT and ICM services within the same team. EBM interventions provided as core component of team function.
Step Down /Graduation	Transfer to less intensive service if demonstrated ability to function during gradual reduction in services over approximately 2 years.	Step down from board into the same team; able to move back onto the FACT board as needed. If stable for 2-3 years can be transferred to lower intensity of care in the local community.	Care coordinators facilitate transfer onto other teams within the same organization or other organizations as client support needs change over time.	Step down from board into the same team; able to move back onto board as needed. If stable for 2-3 years, step down to General Practitioner.
Continuity of care	Some teams may be hospital based. A small number may have psychiatrist with admission privileges. Most are community teams with varying types of relationships and arrangements with local hospitals.	Working in “transmural” integrated hospital /community services model. The team is not only a gatekeeper for the hospital, but also stays in touch with the client during his or her admission and retains the overall coordination of the client’s treatment.	Team part of a community support services organization offering case management, ACT, justice prevention and diversion services, short term residential crisis services and group based services. No established relationships with local hospital inpatient units.	Working in “transmural” integrated hospital /community services model. The FACT team is not only a gatekeeper for the hospital, but also stays in touch with the client during his or her admission and retains the overall coordination of the client’s treatment.

^a^Ministry of Health and Long Term Care, 2004

^b^van Veldhuizen J and Bahler M, 2013

The East team, aiming to integrate hospital with community based expertise and resources, was implemented by adapting a pre-existing urban academic hospital ACT team serving a large homeless population [31]. Co-located with the hospital’s primary care centre, the East team, enhanced by a clinical psychologist, as well as case managers and a personal support worker, focuses on hospital-primary care-community integration and the delivery of evidence-based interventions for a range of mental disorders. Service users, including those previously receiving ACT services as well as new referrals, can access a continuum of ACT and ICM services within the same team, with approximately 50-60% of service users requiring ACT level of support. The South team, implemented by a community mental health organization serving adults with a diversity of needs, was launched as a new team, triaging and assessing new referrals and coordinating their access to a range of pre-existing ICM, ACT, and crisis services in the host organization. Composed of nurses, addiction specialists, social workers, behavioural therapists, and care coordinators, the South team has access to a psychiatric consultant, and provides enhanced ICM support through multidisciplinary assessment and individual case management to service users with complex yet more moderate needs, while facilitating care transitions within the organization’s various programs, including ACT, as support needs change over time.

Both IS teams accept referrals for adults aged 18-65 years (with the South Team additionally accepting youth older than 16 as appropriate) with a variety of health and social needs, including challenges in performing activities of daily living and functioning in the community, housing needs, criminal justice involvement, substance use, and acute or chronic medical illness, including developmental disabilities. The majority of early referrals to the East team were 25-54 years of age (74%), male (57%), had a history of violence/aggression (66%) and a substance use disorder (52%). Approximately 48% had no fixed address. Similarly, the majority of referrals to the South team were 25-54 years of age (73%), male (62%), with a history of self-harm/suicide attempts and psychotic disorders (56%). Approximately 17% had no fixed address.

### Design and data collection

The evaluation included review of program documents (e.g. meeting minutes, program descriptions and policies), and qualitative data collection with a total of 49 stakeholders. We conducted two staff focus groups, three service user focus groups, and seventeen key informant interviews with program and system-level stakeholders. Data collection took place between October 23, 2014 and March 2, 2015.

All staff of the East and South IS teams were invited by the study coordinator to participate in a focus group, exploring staff (*n* = 25) perceptions of the new team based models and the early implementation process, including key program components and staff perspectives on what worked well and what were the challenges during early implementation [see Additional file 1]. Service user participants (*n* = 17) were recruited through convenience sampling. IS staff offered information on the study and directed potential participants to the study coordinator. One service user focus group was conducted with individuals who transitioned from the ACT team to the East team, while the other two focus groups engaged service users newly served by the two IS teams. Service user focus groups elicited information on the services and supports received by their respective teams and their experiences of these services [see Additional file 2]. Key informant interviews (*n* = 17) focused on key program ingredients and program and system-level factors influencing implementation [see Additional file 3]. Key informants were recruited through snowball sampling and included program managers, team leaders, psychiatrists, primary health care providers and relevant decision-makers.

Research staff, not involved in care provision, obtained written informed consent from all participants. Focus groups with staff and program participants lasted approximately 75 min; key informant interviews were approximately one hour in duration. All focus groups and interviews were audio recorded and transcribed verbatim; names and places were anonymized. The study was approved by the Research Ethics Board at St. Michael’s Hospital.

### Analysis

Interview and focus group transcripts were analyzed using thematic analysis, which involves the identification of common themes that span multiple interviews and focus groups [32, 33]. Two researchers independently examined a subset of the transcripts line-by-line, and grouped this qualitative data into codes or threads. They compared their approaches and resolved differences in coding strategies, arriving at a preliminary coding framework with the team’s lead researcher. This coding framework was applied to an additional subset of transcripts and further expanded to accommodate new data. The final coding framework was used by research staff to code all transcripts and program documents. The research team met regularly to review coding categories and reduce them to a smaller number of higher level themes that were internally coherent, consistent and distinctive [32]. Research staff organized member-checking workshops with staff and management from both IS teams to establish the trustworthiness of the data. Analysis was facilitated by Nvivo 10.0 version software.

## Results

Our findings of early implementation drivers are organized using Durlak and DuPre’s conceptual framework [34], including the external context, provider characteristics, model characteristics, program delivery factors and support system factors. Findings support the two team-based models’ success in engaging the target population. Implementation strengths included the broad recognition of existing service gaps, the use of interdisciplinary teams, experienced service providers, partnerships and collaboration across sectors and levels of care, training and team building activities. Emerging challenges included lack of complementary support services such as suitable housing, organizational contexts reluctant to embrace change and risk, and limited service provider and organizational capacity to deliver evidence-based interventions. Some challenges were shared, and others were unique to one or the other IS team as described below.

### External context: system and community factors

#### Facilitator: an identified need for change to address the needs of underserviced populations

As noted earlier, the IS teams were funded by the local health authority, prioritizing adults in the urban core who were poorly served by available services. As one key informant said, “[Ontario] ACT teams have shied away from people that are homeless or have had criminal justice involvement, or developmental disabilities” (KI 16). This service user, in describing his situation, summarized the challenges facing many individuals prior to their referral to an IS team:

“My health was poor. I was sleeping on the street a lot. I was going through starvation periods, where I had no food…I was getting in trouble with the law, assaulting people… hearing voices and seeing things.”

Recognition of the service gaps for this vulnerable population led to dedicated funding and incentives for provider organizations to work across services and sectors, helping launch new partnerships and collaborations: “I think we have to keep pushing our entire care system, to the point where we can really work together on [more] teams that cross organizations” (KI 15).

#### Challenge: Lack of complementary resources and supports

As several study participants identified, the complex challenges in service users’ lives have deep, structural roots that community mental health services cannot address alone. Stakeholders recognized the limitations inherent in challenging the broader factors related to the social determinants of health: “Things like housing, things like a safe neighbourhood, access to quality food – there’s a lot of food insecurity…We’re dealing with issues of poverty…we’re operating within a context of a macro system that doesn’t promote recovery.” Key informant participants further reflected on the implications of this context:

My anxieties were, you know, we are going to be only one team in a fairly hard-to-serve environment…I think there are a lot of systemic problems, that, you know, exist here ... it’s good to have one flexible piece, but I think all pieces need to become flexible. (KI 1).

Team members identified the need for additional flexible resources in the community and systemic change, such as integrated primary care services, direct access to housing and rent supplements for those experiencing homelessness, or interim housing options for those referred upon exit from the criminal justice system.

### Provider characteristics: a collaboration of the willing

#### Facilitator: commitment to learning and improvement

The organizations involved in IS team implementation had prior experience in serving adults with complex health and social needs, in service innovation and inter-sectoral partnerships, and were among few providers interested in exploring new approaches to service provision for this population. Stakeholders and managers at the organizational level were optimistic about the IS teams’ potential for influencing systemic change, and were keen to evaluate their implementation and outcomes: “I do believe that we need more of this type of interventions … than just more case management.” (KI 4).

As implementation progressed, both teams encountered the need for process and practice improvements. Early on, the South team enlisted an external program consultant to engage staff in the development of team processes and protocols. Staff team members appreciated the opportunity to give feedback:

[The program consultant] would then be able to make those adjustments on whether – be it charts of how things would flow through, or whose role was going to be what, or what our care plan was going to look like, or our referral forms… he was able to kind of put that in place.

An East team key informant, similarly noted:

If we’re successful or learn from the process and the system, we can influence the system … we would have, then, a community partner and a hospital who have gained knowledge about how that [hospital community integration] works and could replicate. (KI 5).

#### Challenge: negotiating organizational shifts and change management

The East team transformed a pre-existing hospital based ACT team to develop a flexible approach to service delivery in partnership with a community agency, bringing together hospital and community expertise and resources. In doing so, they encountered challenges and tensions in integrating divergent operational and human resource policies between the partner organizations, including reporting structures, and staff compensation. A key informant reflected, “I think we needed to talk about it from the beginning, I think, and do more things in partnership” (KI 2). Stakeholders also discussed the need to offer adequate time and support for frontline staff during transitions of such scale. Summarizing the views of many frontline providers, one East team staff member said, “Things went so quickly and nobody slowed down … it was so top-down and nobody came to talk to us.”

Another key informant noted the benefits of having staff with a mix of clinical expertise and community knowledge in the hospital led East team:

We brought case managers [from a community mental health organization] who have a really good knowledge of the community, and they bring a different perspective to client care…I’m seeing a really nice blend, because [these case managers] are learning something about the medical model, which is important because our clients do have a lot of medical co-morbidities. (KI 14).

Introducing a flexible, recovery-focused model for individuals with complex mental health needs required large shifts in perspective and service delivery by frontline providers, who were hesitant to embrace new practice requirements. One key informant from the East team (KI 7) noted a gradual “culture shift happening, but I don’t think we’re all the way there.” Some frontline staff members had difficulty adapting to the changes, and this contributed to high staff turnover in the East team during the early phases of implementation.

### Model characteristics: compatibility with local contexts

#### Facilitator: non-diagnostic eligibility criteria and flexible level of support

The IS team’s eligibility criteria enabled many service users to access care that has previously been denied to them. In the words of a service user: “I’ve been in the healthcare and mental healthcare system for many years…I just was continually falling through the cracks. [The IS team] was instant in getting me connected.” Staff felt that the new teams allowed for the possibility of “fitting the model to the patient” rather than “fitting the patient to the model”, thus reducing barriers to access and allowing for more timely and appropriate care. The teams aimed to work with adults who had, “not necessarily the diagnosis, but just [the] presentation” (KI 12). One provider stated:

I think as time passes we’re looking less and less about you’ve got to meet A, B, C, D criteria... It’s more, “Okay, you’re very difficult to serve. Nobody else has been able to provide the services that you need. Maybe you ‘re appropriate for an IS team.

The ability to titrate the level of support over time was seen as necessary and valuable: “Not everyone needs that [ACT team] level of intensity, but at some point, most people do” (KI 4). A stakeholder with the East team described the potential for movement within the team, while noting this movement was still in its early stages:

We now have to start to see the flow and the movement on the other end… They are, you know, ACT clients we’ve seen every day, sometimes multiple times a day…and then, hopefully, they experience that period of stability. They move over to ICM case management. They are seen once a week …and now, you know, we are actively looking at the discharge planning for people and we’re moving them through that program and that system. (KI 3).

#### Challenge: balancing support needs with a recovery focus

Particularly in the initial stages of implementation, the exclusive focus on adults with complex needs resulted in a caseload with a large number of service users requiring high-intensity services. Staff suggested that “one client from the IS team would probably be equivalent to maybe three on an ACT team, in terms of management”. Some staff felt this workload was not conducive to the recovery-focused service they wanted to provide:

We are supposed to be challenging discrimination in a recovery model but oftentimes what happens is that we just don’t have the time to do that, so we just move on.

As another staff member described, “you are running around with your little garden hose trying to put out a forest fire.” A key informant with the East team said, “I would have hoped to have a true FACT team, where… you have a mix of, you know, high needs, moderate needs, and low needs.” Instead, this person felt the model, at least in its early stage, was more of a “super-ACT team… many too-complex clients, which makes it difficult for staff” (KI 16). Similarly, South team members explained the challenges with their case management approach: “Speaking on behalf of the nurses, we have other responsibilities for all the clients, not just our own caseload”; and, “I may have an entire caseload of people who require med observes, whereas somebody else doesn’t have any” (KI 11). Despite having access to other ICM and ACT services within the organization, staff and stakeholders of this team recommended moving towards a “more ACT-like model”, with more shared caseloads, similar to the East team, to distribute the work more equitably and facilitate recovery focused and evidence based care.

### Program delivery system: organizational capacity

#### Facilitator: early and ongoing communication with key partner organizations

Both teams invested in relationship building and regular communication with key hospital and community organizations to ensure input, transparency and accountability. The hospital led East team described “a couple of meetings with all service organizations” in the geographical area, where the team would report on, “Here’s where we are, here’s the draft [eligibility] criteria at this point” (KI 7). This ensured that the program adaptations would address local service gaps, as well as “build positive relationships for the referral process” (KI 14). Likewise, community organization led South team staff described “working really diligently at making these connections”. As a result, “they see us as being a really clean sort of resource and referral point” (South team staff). Stakeholders also noted that with more community outreach, working collaboratively with other agencies, the easier it would be for the teams to coordinate care for those receiving services from multiple agencies. One key informant emphasized that creating collaborative service user care plans with other agencies “provides an opportunity to engage with a range of other healthcare providers … which then helps to create a culture spread around coordinated care planning to primary care, to community mental health, etc.” (KI7).

#### Challenge: coordination with acute care resources

Often, the complexity of service user needs exceeded the IS team’s or host community organization’s expertise and resources. Both teams quickly recognized that management of medical conditions was particularly important for this population, facilitated in the East team by colocation with primary care services. Furthermore, service users frequently required acute psychiatric and medical care. Accessing hospital care and creating appropriate discharge plans could be challenging, especially for the South team, where the local hospital was not a direct partner in service delivery. One South team staff member summarized, “It’s hard as a community team who services this clientele with these complexities to not have any type of say or privileges for admitting in, in hospitals.” As one key informant explained, “What you don’t want happening, and what happens sometimes is, ‘We’re going to discharge Tom on Thursday.’ On Friday you get a call – ‘Oh, well, actually we discharged him on Wednesday’” (KI 15). The South team saw opportunities for better communication when service users required hospitalization:

It would be really interesting to have a community person in the hospital that would kind of filter and control the information within the hospital system. Somebody consistent we could actually go to, to provide the information and feedback to. Almost like a back door. (South team staff).

### Program support system: training and technical assistance

#### Facilitator: staff training and team building

Training on evidence-based practices such as Motivational Interviewing was provided to team members prior to service launch. This was perceived as helpful not only for supporting service users, but for building team cohesion. Speaking of their experience with full-team, intensive training on Dialectical Behavioural Therapy (DBT), a South team staff member noted,

I think it might contribute to the functioning of the team, because every single week we’re reminded to be thinking dialectically. We are reminded to be working in this kind of approach…and so, I can’t help but think that is influencing how we don’t just engage with our clients, but how engage with ourselves.

#### Challenge: supporting delivery of evidence-based approaches

Participants from both teams noted the need for ongoing training and supervision to ensure consistent delivery of evidence-based practices. As one key informant said,

I think we do need, as a team, to get much more training in terms of how do we support clients in their recovery. I mean, I think theoretically we all know it, but I’m not so sure that we know it that much – how does it really play out in real life? How do we actually do that? (KI 14).

Stakeholders identified that training for all staff may be difficult to provide when simultaneously caring for an active caseload, but stressed the importance of building in time and resources for individual and team training and supervision, facilitated in the East team by team based psychiatrists and a clinical psychologist.

## Discussion

Our findings suggest that it is possible to increase diagnostic and service flexibility of community mental health teams in response to service access barriers for individuals with complex health and social needs. In Toronto, Canada’s largest urban centre, the implementation of novel approaches to serving this population was facilitated by recognition of the need for program adaptations and improvements to address existing service gaps, of the need for effective change management, as well as commitment and capacity to deliver flexible, multidisciplinary approaches to care. Although participating organizations were early champions of the need for improvement, our findings exposed significant provider, team and organizational challenges that need to be overcome in transformational efforts of this magnitude.

Community and political contexts can have large impacts on the implementation of health services [34–36]: our findings suggest that recognition of service gaps by both local health authorities and provider organizations facilitated the introduction and acceptability of the new teams. In supporting change and innovation, participants echoed concerns raised in many jurisdictions regarding service user tenure within high intensity teams, leading to services that quickly reach capacity and may not facilitate the provision of appropriate levels of care [8, 37]. Service providers and key informants also stressed the limitations of these services within inadequately resourced mental health service delivery systems, or without broader systemic change addressing lack of housing and adequate income supports for adults experiencing severe disabilities. These findings emphasize the importance of stakeholder engagement and of local needs assessment to assess readiness for change and implementation of new service approaches for this population.

The design and philosophies of the teams, and the importance placed by staff and stakeholders on the flexibility and adaptability of the models, as well as the competencies of frontline providers to deliver evidence based interventions echo findings from research on successful implementation of health care practices, including Assertive Community Treatment [26, 29, 30, 34, 35]. However, research has also identified that belief in, and the design of an intervention is not enough for successful implementation: “practices must be implemented actively” [26]. Initial and ongoing commitment to the development of community partnerships and inclusion of staff in decision-making facilitated the implementation of responsive and appropriate services. A study on key domains of successful implementation in community mental health suggested that engaged leaders can identify and put forth specific strategies to lead active implementation, such as redesigning workplace policies and adapting staff’s assigned duties [26]. Our findings indicate instances where this was occurring within the IS teams, such as bringing in an external consultant to engage staff in service design, and providing full team training in evidence-based practices. The study also identified places in need of further attention, including the balancing of case management caseloads, revisiting operational policies and reporting structures in multi-agency service teams, providing ongoing training opportunities in team-building formats, and, for services delivered by community organizations, developing stronger partnerships with acute care facilities. Our findings have parallels with those in other contexts, and highlight the importance of effective team function and clinical leadership [29, 30].

Dedicating time and resources to these aspects of implementation may also improve staff capacity and comfort with delivering care to adults with complex needs, potentially preventing turnover and encouraging a work climate more open to change and innovation, mitigating the risk of burn out and emotional exhaustion that might result from inadequate training or resources [29]. Organizational support and leadership may be particularly critical when a new team is developed by transforming a previous program, such as the East team’s transition from ACT. Positive service user outcomes from a similar ACT-to-FACT process in the UK suggest that growing pains and higher staff to service user ratios have not had a negative effect on service provision [12, 17], and a recent study highlighted positive mental health professional experiences of the FACT model [38], though, as others have noted, further evaluation of these approaches is needed [39–41]. As staff from the South team noted, adopting more “ACT-like” approaches by ICM teams by broader adoption of team based approaches to care and assumption of clinical responsibility across hospital and community settings may well be key ingredients of successfully engaging and supporting the target population.

Despite the use of rigorous qualitative methods, this research is limited in its generalizability due to the local service context in Toronto, Canada. Additionally, this study was completed during the early phase of program implementation, suggesting that examination of later phases of implementation and sustainability may be warranted, including purposive sampling of service user participants to increase trustworthiness of the data. In these early phases of program implementation, service user participation was limited to a convenience sample, introducing selection bias for this stakeholder group, and participant check in was not pursued with service user participants, given the focus of the study at this stage. Despite the limitations above, and the limitations of qualitative research in general, including researcher subjectivity in analysis and interpretation of data, our findings are relevant to many jurisdictions facing similar challenges, and may be helpful in efforts to innovate within existing community mental health models.

## Conclusions

Stakeholders with a range of expertise and experiences offer important perspectives on the acceptability and implementation drivers of flexible models of service delivery for adults with complex health and social needs. Lessons learned can guide continued improvement in community mental health services and call for rigorous research to establish the effectiveness of novel interventions.

## Additional files

Additional file 1: Appendix X_Guide_Staff Focus Group Discussion Guide. (DOC 48 kb)

Additional file 2: Appendix Y_Guide_Client Focus Group Discussion Guide. (DOC 49 kb)

Additional file 3: Appendix W_Guide_Key Informant Interview Guide. (DOC 47 kb)

## Abbreviations

ACT: Assertive Community Treatment
FACT: Flexible Assertive Community Treatment
ICM: Intensive Case Management
IS: Integrated Service
SMI: Serious Mental Illness
