# Supplementary material for: Building flexibility and managing complexity in community mental health: lessons learned in a large urban centre
Source: BMC Psychiatry. 2018 Jan 24;18:20. doi: 10.1186/s12888-018-1597-y (PMC5784615; doi:10.1186/s12888-018-1597-y)
Supplement: Supplementary file 1 — Appendix X_Guide_Staff Focus Group Discussion Guide. (DOC 48 kb) [file 12888_2018_1597_MOESM1_ESM.doc]

**APPENDIX X: STAFF FOCUS GROUP DISCUSSION GUIDE**

We are interested in learning from your experience working with the East/South Team and discussing some of the successes and challenges so far. We will have a few questions about how the program was designed, and then we have a few questions about how the program is working so far.

**How the program was designed –** (understanding the program model)

To start, let’s talk a bit about the team and how it was designed.

1. Can you please describe how the team works?

- What is the role of the team? What is your role?
- What is the target client population?

1. What are the key services provided by the team?
2. What are the key components or ingredients of the team?

- Staffing? Training? Process for working with clients?

1. What are the primary goals of the team?

- What are the goals for how the team works with clients?
- What are the goals for how the team works together?
- What are the goals for how the team works with other organizations?

1. How will the services and components of the team lead to changes for clients?
2. What is the role of the team in integrating hospital, transitional, and community services?

- What is the relationship of the team to: The Central Access Point for mental health services? Other referral sources? The local health authority?

1. Based on your experience, what would you recommend to another organization that was trying to serve clients with complex mental health needs?

**How the program was implemented** – (how the program is working so far)

Thanks for sharing your thoughts on those questions. Now, let’s move on and think about how the program is working so far, and how it has been developing since it was first implemented.

1. What is working well in terms of the program implementation?

- What factors have helped the implementation of the program to go smoothly?
- How have those factors impacted the ability to meet the goals of the program?

1. What is not working well in terms of the program implementation?

- What challenges and barriers have emerged as the program has been implemented?
- How have those factors impacted the ability to meet the goals of the program?

1. What are the key factors in the program’s environment that are influencing the program implementation?
2. What are the key relationships that are influencing the program implementation?

- Network of services, community and stakeholders?
- Organizations that refer to the team?
- Services that the team refers to or collaborates with?

1. What are the emerging strengths of the program model?

- How is this program model different from others in the community?

1. What are the emerging weaknesses of the program model that may need attention in the future?

- How is this program model different from others in the community?

1. How is the program working well for specific types of clients?
2. How is the program not working well for specific types of clients?
3. How has the program had to adapt to changes in the environment since implementation?

- What new program innovations have resulted from changes in the environment?
- How has the program model changed since its conception?
- How has the program model changed since its launch?

1. Based on your experience, what would you recommend to another organization that was trying to implement services for people with complex mental health and social needs?

- What would you do the same? What would you do differently?
- How would you embed the program into the network of existing services?

Do you have any questions for me?

Thank you very much for your participation today. We appreciate your willingness to share your experiences.

I am going to turn the tape recorder off now.
