# Supplementary material for: Building flexibility and managing complexity in community mental health: lessons learned in a large urban centre
Source: BMC Psychiatry. 2018 Jan 24;18:20. doi: 10.1186/s12888-018-1597-y (PMC5784615; doi:10.1186/s12888-018-1597-y)
Supplement: Supplementary file 2 — Appendix Y_Guide_Client Focus Group Discussion Guide. (DOC 49 kb) [file 12888_2018_1597_MOESM2_ESM.doc]

**APPENDIX Y: CLIENT FOCUS GROUP DISCUSSION GUIDE**

We are interested in learning from your experience working with the South/East Team and discussing some of the successes and challenges so far. We will have a few questions about how your experience as a client so far, and then we have a few questions about what you think the strengths and weaknesses of the team are so far.

**To start, let’s talk a bit about the time before you joined the team…**

1. What services, if any, were you using before you started working with the East/South team?
2. How would you describe your health, wellbeing and personal circumstances before you started working with the East/South Team?

**Since you have been working with the Team…**

1. What services have you been receiving from the team?

- From your primary case manager?
- From the psychiatrist?
- From other members of the team – nurses, psychologists?
- From other programs at St. Michael's Hospital/Reconnect?

1. What does a typical day working with the team look like for you?

- How do you work with the team?
  - Do you usually see multiple people, or just the same person all the time?
  - Home visits or office visits?

1. If you’re having a challenging day, what do you do? Who do you contact?
2. If you were hospitalized since joining the team, how if at all, was the team involved?
3. How has working with the team helped you meet your goals (health, housing, employment, education)?

- Probes: helped you to… meet your needs? Manage your health?

1. How has working with the team influenced your life, for better or for worse?
2. Are there any areas in which the team has not been able to help you?
3. What are some of the strengths of the team?

- What do you like best about working with the team?

1. What are some of the weaknesses of the team that could be improved?

- What do you like least about working with the team?
- What do you think the solutions could be?

1. How, if at all, is the Eat/South team different from other programs in the community?
2. Based on your experience, what would you recommend if you were designing a new team like the East/West Team?

- What would you keep? What would you change or do differently?

Do you have any questions for me?

Thank you very much for your participation today. We appreciate your willingness to share your experiences.

I am going to turn the tape recorder off now.
