# Supplementary material for: Building flexibility and managing complexity in community mental health: lessons learned in a large urban centre
Source: BMC Psychiatry. 2018 Jan 24;18:20. doi: 10.1186/s12888-018-1597-y (PMC5784615; doi:10.1186/s12888-018-1597-y)
Supplement: Supplementary file 3 — Appendix W_Guide_Key Informant Interview Guide. (DOC 47 kb) [file 12888_2018_1597_MOESM3_ESM.doc]

**APPENDIX W: KEY INFORMANT INTERVIEW GUIDE**

The following interview questions will be most applicable for program managers or directors from a mental health service provider or organization that works with one of the Integrated Service Coordination Teams, or a community stakeholder. For key informant interviews with program managers or team leads from one of the Integrated Service Coordination Teams, the questions will be more similar to the Staff Focus Group Discussion Guide (please see Appendix X)

We are interested in learning from your experience working with the South and East teams and discussing some of the successes and challenges so far. We have a few questions about how your organization works with these teams, and then we will have a few questions about how you think they are working so far.

**Program Model**

To start…

1. Can you please describe your organization, and how it works with the South and East teams?

- What are the primary goals of your organization?
- How does working with the two teams relate to your organization’s goals?

Based on your understanding of how the 2 teams were designed…

1. What are the primary goals of these teams?
2. How will the services and components of the these teams lead to changes for clients? Changes for other service providers? Changes for the mental health system?
3. How are these teams different from other programs?

Thinking about these teams and other organizations that provide services to people with complex mental health needs…

1. How can we better integrate care for people with complex mental health needs?

- What is the role of the South and East teams?
- How are these teams working with the central access point for mental health services, other programs, or the local health authority to integrate care?

1. What are the key-evidence based interventions for people with complex mental health needs?

- How are teams working to deliver evidence-based interventions?
- What else is needed to deliver evidence-based interventions?

1. Based on your experience, what would you recommend to another organization that was trying to adapt their services to better serve those with complex mental health and social needs??

**Program Implementation**

Thank you for sharing your thoughts on those questions. Now, we have a few questions about how you think the south and East teams are working so far, and how they have been developing since they were implemented.

1. What is working well in terms of the program implementation?

- What is working well in the way that your organization works with these teams?

1. What is not working well in terms of the program implementation?

- What could be improved in the way that your organization works with the teams?

1. Based on your experience, what would you recommend to another organization that was trying to implement services for people with complex mental health and social needs?

- What would you do the same? What would you do differently?
- How would you embed the program into the network of existing services?

Do you have any questions for me?

Thank you very much for your participation today. We appreciate your willingness to share your experiences.

I am going to turn the tape recorder off now.

Are there other stakeholders that you think we should interview, to learn about Integrated Service Coordination within the Mid East Toronto and South Toronto Health Links?
